# Trans-kingdom Cross-Talk: Small RNAs on the Move

**DOI:** 10.1371/journal.pgen.1004602

**Published:** 2014-09-04

**Authors:** Marijn Knip, Maria E. Constantin, Hans Thordal-Christensen

**Affiliations:** Department of Plant and Environmental Sciences, University of Copenhagen, Frederiksberg, Denmark; University of Utah School of Medicine, United States of America

## Abstract

This review focuses on the mobility of small RNA (sRNA) molecules from the perspective of trans-kingdom gene silencing. Mobility of sRNA molecules within organisms is a well-known phenomenon, facilitating gene silencing between cells and tissues. sRNA signals are also transmitted between organisms of the same species and of different species. Remarkably, in recent years many examples of RNA-signal exchange have been described to occur between organisms of different kingdoms. These examples are predominantly found in interactions between hosts and their pathogens, parasites, and symbionts. However, they may only represent the tip of the iceberg, since the emerging picture suggests that organisms in biological niches commonly exchange RNA-silencing signals. In this case, we need to take this into account fully to understand how a given biological equilibrium is obtained. Despite many observations of trans-kingdom RNA signal transfer, several mechanistic aspects of these signals remain unknown. Such RNA signal transfer is already being exploited for practical purposes, though. Pathogen genes can be silenced by plant-produced sRNAs designed to affect these genes. This is also known as Host-Induced Genes Silencing (HIGS), and it has the potential to become an important disease-control method in the future.

## Introduction

Since the discovery of gene silencing induced by inverse transcripts in the 1980s [1] and Fire and Mello's discovery in 1998 that double-stranded RNA (dsRNA) can activate gene silencing in *Caenorhabditis elegans* [2], our understanding of the complex role of RNA in gene regulation has increased considerably. Different types of small RNA (sRNA) molecules have been identified over the years, of which microRNAs (miRNAs) and small-interfering RNAs (siRNAs) are the main types. In this review, we will primarily use the shared term, sRNA, and generally not distinguish between miRNAs and siRNAs.

sRNAs are typically 19–25 nt long, and they are produced from larger dsRNA or hairpin RNA (hpRNA) molecules by DICER (DCR) or DICER-like (DCL) proteins. They bind to complementary mRNA targets with the help of an Argonaute (AGO) protein, leading to transcriptional and post-transcriptional gene silencing. This complex of an sRNA and an AGO protein is called the RNA-Induced Silencing Complex (RISC). The components of the RNA silencing machinery are widely conserved in eukaryotes (reviewed in [3]–[5]). sRNA-guided transcriptional gene silencing in the nucleus involves RNA polymerase II release, epigenetic histone modifications, typically introducing the H3K9 methylation mark, and DNA methylation. Post-transcriptional gene silencing in the cytosol involves mRNA cleavage and inhibition of translation (reviewed in [3], [6], [7]). These modes of sRNA-guided gene silencing are often referred to as RNA interference (RNAi). The essential components of the RNAi mechanism appear to have been present in the last eukaryotic common ancestor, although species in several super-groups of the eukaryotic tree seem to have lost components of the RNAi machinery independently. These include *Saccharomyces cerevisiae* (Unikonta), *Trypanosoma cruzi* and *Leishmania major* (Excavata), *Cyanidioschyzon merolae* (Archaeplastida), and *Plasmodium falciparum* (Chromalveolata) [8], [9]. It is widely believed that RNAi evolved as a measure to control viruses and transposable elements. However, as we review here, RNAi also functions in communication between hosts and more advanced pathogens and parasites. Otherwise, it has come to play essential roles in gene regulation important for endogenous life processes, including fine-tuning of mechanisms for innate immunity [10].

**Figure 1:**
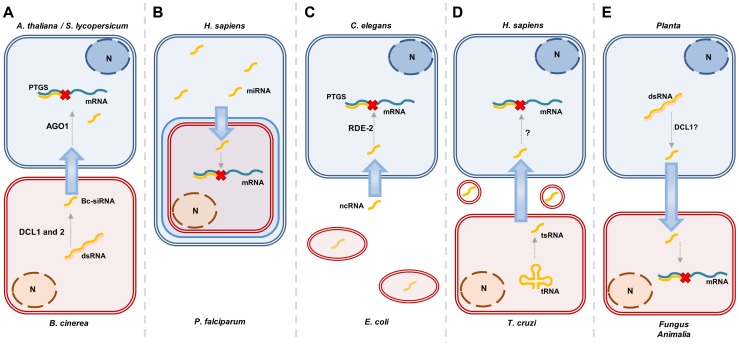
Overview of different situations in which sRNA transfer occurs. **A,** *Botrytis cinerea* can transfer Bc-siRNA to its host. This process has been shown to be dependent on AGO1 in the host, *Arabidopsis thaliana* and on both Dcl1 and 2 in *Botrytis cinerea* [23]. **B,** Human miRNAs can be translocated to the malaria-parasite, *P. falciparum*, where they interfere with translation [16]. **C,** The nematode *C. elegans* has been shown to take up *E. coli*-produced ncRNAs that subsequently influence their foraging behavior. This is dependent on the *C. elegans* protein RDE-2, that is essential for RNAi [17]. **D,** The Chagas disease-causing parasite, *T cruzi*, produces tRNA-derived sRNAs (tsRNAs) that are exported from the cell in vesicles. These vesicles are shown to increase infectability of host cells, suggesting this might be caused by the tsRNAs. This has not been shown directly though [14]. **E,** The expression of sRNA-generating constructs to silence genes in pathogens, or other closely associated species, has now been demonstrated for many species combinations. This process is suggested to be dependent on Dcl1, since Dcl2, 3, and 4 seem to be dispensable to induce silencing by an Arabidopsis-expressed hairpin in the insect, *Helicoverpa armigera* [24].

RNA molecules have been found to be mobile within organisms, and numerous cases in which RNA-silencing signals travel between different organisms have now been described. These organisms can be of the same species, where breast-feeding of infants may provide an example of RNA-mediated gene regulation [11], or of different species, for instance between plants parasitized by other plants [12]. In recent years, both animals and plants have been found to exchange sRNA with closely interacting pathogenic, parasitic, or symbiotic organisms [13]–[15]. Trans-kingdom movement of RNA-silencing signals has been reported to occur between a wide range of species: from humans to the malaria-causing chromist, *P. falciparum* [16], from bacteria to nematodes [17], from plants to pathogenic and symbiotic microbes [18]–[21], from plants to nematodes [22], from fungal pathogens to plants [23], and from plants to insects [24]. These examples are detailed in Figure 1 and Table 1. The method of Host-Induced Gene Silencing (HIGS) exploits the silencing effect of sRNA signals in interacting organisms, and involves host expression of sRNA-generating constructs directed against genes in associated pathogens, parasites, or symbionts [18]–[20], [22], [24]–[28].

**Table 1:** Examples of trans-kingdom RNA-mediated signal transfer.

Species				Experimental evidence	Ref.
From	K	To	K		
*Homo sapiens*	A	*Plasmodium falciparum*	C	Detection of miRNA and annealing to mRNA in target-species	[16]
*Trypanosoma cruzi*	Pr	*Homo sapiens*	A	Detection of sRNAs in extracellular vesicles. No direct evidence for sRNA effect, but vesicles induce effect.	[14]
*Botrytis cinerea*	F	*Arabidopsis thaliana, Solanum lycopersicum*	P	Detection of sRNAs and down-regulation of their targets in the host species. Hijack of RNAi machinery by fungal sRNAs.	[23]
*Triticum aestivum*	P	*Puccinia triticina*	F	RNA produced from RNA virus in planta leads to gene down-regulation in target species	[62]
*Hordeum vulgare*	P	*Blumeria graminis*	F	Hairpin expression in planta leads to gene down-regulation in target species	[18]
*Medicago truncatula*	P	*Glomus intraradices*	F	Hairpin expression in planta leads to gene down-regulation in target species	[21]
*Musa paradisiaca*	P	*Fusarium oxysporum*	F	Phenotype of fungus grown in vitro on medium containing sRNA. Hairpin expression in planta leads to gene down-regulation in target species	[20]
*Arabidopsis thaliana*	P	*Fusarium graminearum*	F	Hairpin expression in planta leads to gene down-regulation in target species and suppresses fungal growth	[19]
*Nicotiana tabacum*	P	*Phytophtora capsici*	C	Hairpin expression in planta leads to gene down-regulation in target species	[28]
*Glycine max*	P	*Meloidogyne incognita*	A	Hairpin expression in planta leads to gene down-regulation in target species	[22]
*Arabidopsis thaliana, Nicothiana benthamiana*	P	*Helicoverpa armigera*	A	Hairpin expression in planta leads to gene down-regulation in target species	[24]
*Zea mays*	P	*Diabrotica virgifera* and other coleopteran spp.	A	Hairpin expression in planta leads to gene down-regulation in target species	[30]
*Arabidopsis thaliana*	P	*Myzus persicae*	A	Hairpin expression in planta leads to gene down-regulation in target species	[63]
*Escherichia coli*	B	*Caenorhabditis elegans*	A	Bacterial ncRNAs down-regulate genes and alter nematode behavior	[17]

The “From” and “To” columns indicate the direction of the reported signals. The “K” column shows the kingdom in which the organisms are classified. Although more advanced classifications of the tree of life have been proposed, we chose to use the six kingdom system proposed by Cavalier-Smith in 1998 [64]. P, Planta; A, Animalia; F, Fungi; B, Bacteria; C, Chromists; Pr, Protists.

Many aspects of these trans-kingdom silencing phenomena remain poorly understood. These include how specific sRNAs are selected for transport, how sRNAs are transported outside the cell, the way they recognize and enter their target cell, and the mode by which these sRNAs use the target cells' RNAi machinery to convey their silencing effect. Here, we will deliberate on the mechanisms that could be involved in the transfer of these silencing signals and address some of the many questions surrounding the intriguing phenomenon of trans-kingdom sRNA mobility.

## The Biological Context of RNA Trans-kingdom Transfer

The examples in Table 1 and Figure 1 suggest that there is a framework, widely conserved in eukaryotes, that allows production, transfer, and perception of RNA signals between very distantly related organisms across the branches of different kingdoms of the tree of life. In the HIGS examples, sRNA-producing constructs are designed to target genes in the interacting organisms, often of different kingdoms. However, evidence is available that natural, endogenous sRNA also target genes in a trans-kingdom manner. For instance, the plant pathogen, *Botrytis cinerea*, exploits siRNAs to target defense genes in Arabidopsis and tomato, thereby enhancing its pathogenicity (Figure 1A) [23]. Another example of this comes from human erythrocytes that use miRNAs to target *P. falciparum* genes and thereby counteract malaria (Figure 1B) [16]. This indicates at the same time that sRNA signaling can be transmitted in both directions between host and invader. Similarly, the parasitic flatworm, *Schistosoma japonicum*, was found to produce miRNAs that could be retrieved from the plasma of rabbits that host it, but it is not clear whether this miRNA has a function in rabbits [15].

Through evolution, hosts and their invaders have undergone amazing arms races involving appearances of receptors and downstream response mechanisms for detection and defense on the host side, and, e.g., defense suppressing effectors on the side of the invaders. Hitherto, the interactions are described to be based on transfer of proteins and low-molecular-weight molecules between the organisms. However, the results of LaMonte et al. [16] and Weiberg et al. [23] indicate that RNA can be added to this list of communication molecules.

Even though the occurrence of RNA signal transfer is widespread, it is not surprising that there are organisms that may not be influenced by incoming RNA. The oomycete plant pathogen *Phytophthora parasitica* appears not to be sensitive to sRNA coming from the plant host [29], even though the closely related *Phytophthora capsici* is [28]. If this distinction can be confirmed, it would be very interesting to determine what fundamental difference could account for the susceptibility to exogenous sRNA molecules in one and not the other *Phytophthora* species. This could potentially reveal an essential mechanism of sRNA transfer or RNAi, which would suggest that *P. parasitica*, by being insensitive, has added another level to the molecular arms race between host and pathogens.

The HIGS method provides us with a potential means to decrease the success rate of pathogens and parasites. This can be achieved by engineering host-produced sRNAs to silence essential pathogen transcripts, which under laboratory conditions has been documented to be very efficient [19], [22], [24]. It will be interesting to see how efficient and durable this will be under conditions outside the laboratory. Another way of obtaining host resistance may be based on the fact that pathogens and perhaps parasites also make use of sRNAs in the interaction with hosts. Therefore, the host genes targeted by them could be re-coded to make them insensitive.

## Considerations When Assessing Inter-specific sRNA Transfer

As listed in Table 1 and Figure 1, many species have now been suggested to exchange sRNA signals. However, several of these examples are largely based on correlated phenotypic effects in the target organism after expression of an sRNA-generating construct in the interacting organism (e.g., [20], [27], [30]), and direct evidence for sRNA functioning in the target organism is not given. One reason is the difficulty of detecting the sRNA molecule specifically in the target organism without risk of contamination from the transmitting organism. However, the example of Tinoco et al. [26] offers convincing evidence. Here, GUS enzyme activity was reduced in a transgenic *Fusarium verticillioides* strain after it had attacked a tobacco host plant expressing a *GUS* hairpin construct. The observation was made during in vitro cultivation after the fungus had been recovered from the plant and occurred together with reduced *GUS* transcript level and presence of a *GUS* sRNA in the fungus, the latter detected by northern blot. Furthermore, it was noteworthy that this *GUS* gene silencing could last for an extended period of in vitro growth, i.e., in absence of hp*GUS* from tobacco, while subsequently resuming initial GUS expression levels. In vitro cultivation of one of the two organisms following the interaction overcomes the obvious contamination problem when determining presence of transferred sRNA. Weiberg et al. [23] study the plant RNAi machinery to support the hypothesis that the fungal-induced plant gene suppression indeed is caused by the fungus sRNA functioning in the plant. Plant RNAi in general is required for fungal resistance, and by knocking out *DCL1*, Weiberg et al. show that this also is the case for *B. cinerea*. However, knocking out *AGO1* has the opposite effect on *B. cinerea*, even though these two components are on the same RNAi pathway. This supports the idea that plant AGO1 is used by the fungal sRNA in host gene silencing.

## Alternative Mechanisms of Gene Silencing

In most described instances, both species involved in the exchange of sRNA possess the canonical RNAi machinery. However, trans-kingdom RNA silencing does not necessarily require this. The malaria parasite, which receives human miRNA [16], does not possess homologues of AGO and DCR proteins [31]. The translocated miRNAs were instead found to form chimeric dsRNAs with *P. falciparum* transcripts, thereby inhibiting translation (Figure 1B) [16]. It has been found that the Chagas disease parasite *T. cruzi*, although it also lacks components of the canonical RNAi pathway, produces vesicles that are loaded with both tRNA-derived sRNAs and an Argonaute protein. It seems likely, but has not been directly shown, that these signals could influence host gene expression (Figure 1D) [14]. Gene expression of the nematode, *C. elegans*, can be influenced by non-coding RNA produced by the bacterium *Escherichia coli*. This RNA is being taken up and feeds into the RNAi machinery of the worms, down-regulating the *che-2* gene, which impairs their ability to find food (Figure 1C) [17]. Future studies will show how common such alternative mechanisms are compared to classical RNAi mechanisms.

## Extracellular Transport of sRNA

**Figure 2:**
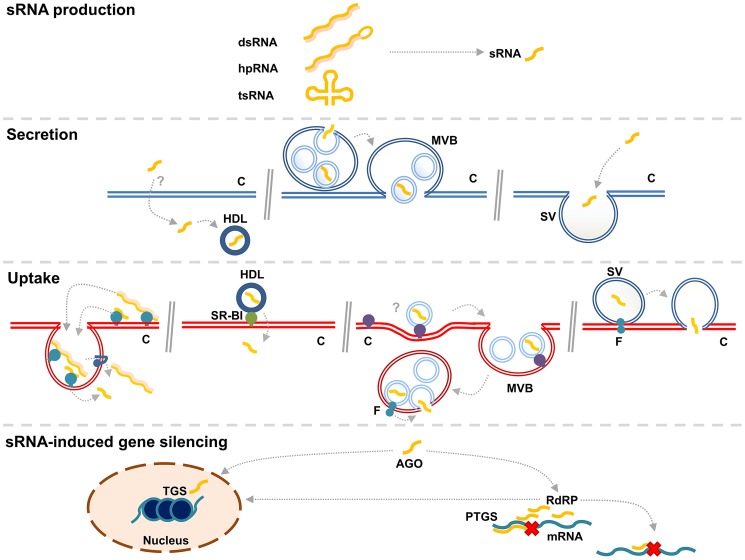
A generalized overview of RNA transfer from one cell (blue) to another (red). sRNA is produced by Dicing of larger dsRNA molecules in the transmitting cell. On the left, non-vesicular dsRNA and sRNA are secreted by unknown mechanisms. Uptake of this RNA is depicted in a manner that resembles SID-1/SID-2 mediated uptake [39]. DsRNA is bound by a receptor and internalized, after which it is taken up into the cytosol by a transmembrane channel, such as SID-1. In the middle, transfer of sRNAs through MVB-mediated exosomes is depicted. A model for loading of sRNA into intraluminal vesicles of MVBs (**MVB**) is suggested [49]. These vesicles are released in the intercellular space as exosomes after fusion of MVBs with the plasma membrane (**PM**). Exosomes are taken up by endocytosis into the receiving cell. It is unknown how sRNA is released into the cytosol, but one could envisage a fusogenic protein (**F**) to be involved, which facilitates fusion of the endosomal and exosomal membranes. On the right, transfer of sRNA in shedding vesicles (**SV**), which are generated directly from the PM, is depicted. How RNA is loaded into SV is unknown. The recipient cell takes up the sRNA after fusion of the SV with the PM in a process that requires fusogenic proteins. SVs might be taken up in an endocytosis-dependent manner and exosomes might be taken up in a membrane fusion event. In the cytosol of the recipient cell, the sRNA is recognized by the RNAi machinery and triggers gene silencing, either through post-transcriptional gene silencing (**PTGS**) or transcriptional gene silencing (**TGS**). During PTGS, amplification of the sRNA signal is provided by RNA-dependent RNA polymerases (**RdRP**), which give rise to secondary sRNAs that can target the same or other transcripts.

With the exception of the situation for intracellular symbionts and pathogens, e.g., *P. falciparum*, a prerequisite for cross-species RNA signaling is extracellular mobility of RNA. Many organisms have been shown to contain extracellular sRNA and several distinct forms of sRNAs have now been found to be mobile in different organisms. We believe that RNA signals that travel between organisms rely on similar mechanisms as those observed for extracellular transport within an organism (Figure 2). In humans, sRNAs have been found to be present in extracellular fluids. This is a hostile environment for RNA, which needs to be protected from degradation. Exported sRNA has been found inside extracellular vesicles and in association with High-Density Lipoprotein (HDL) cholesterol particles (Figure 2) [32]–[34]. How sRNAs are selected for extracellular transport is currently not clear, but the profile of exported sRNA appears to be different from the population of cellular sRNA. This suggests an active selection process [32], [35], [36].

### Non-vesicular extracellular sRNA

As indicated, sRNA not enveloped in a membrane can be found in the extracellular space. It is still unknown how this free sRNA is secreted, not to mention how it is selected for secretion. However, outside the cells it can be associated with HDL and proteins, such as AGO [34], [37]. Ideas of how free sRNA might be taken up by target cells come from studies in *C. elegans*, which revealed key components for uptake of free extracellular dsRNA. These are the transmembrane protein channel, Systemic RNAi-Deficient (SID)-1 [38], and the single-pass transmembrane receptor, SID-2 [39]. It is thought that free dsRNA is internalized from the intestinal lumen by a SID-2–receptor mediated endocytosis, after which dsRNA can escape from the endosome into the cytoplasm by SID-1 (Figure 2) [39]. Unlike SID-1, which is conserved in animals, SID-2 is poorly conserved [40]. An alternative to this protein seems to be scavenger receptors mediating clathrin-dependent endocytosis of cholesterol-conjugated lipoprotein. In cultured human hepatocytes, extracellularly applied cholesterol-conjugated lipoprotein-associated sRNA has been found to be able to induce RNAi [41]. This and other examples indicate that scavenger receptors are required for RNA uptake [34], [41], [42].

### Vesicular extracellular sRNA

RNA in extracellular vesicles has attracted increasing interest as a means of intercellular communication. When first discovered, extracellular vesicles were merely considered to result from stressed cells shedding waste products [43]. However, after the discovery of nucleic acid sequences in these vesicles, they were considered much more interesting, as this suggested that they might facilitate genetic signaling [32]. sRNA-containing vesicles in human plasma are either shedding vesicles, formed by outward-budding at the plasma membrane, or exosomes, formed by inward budding of intraluminal vesicles (ILV) at endosomal membranes of multi-vesicular bodies (MVBs). ILV formation is generally considered to require the Endosomal Sorting Complexes Required for Transport (ESCRT) machinery. The exosomes are subsequently released into the environment when MVBs fuse with the plasma membrane [44]. A subset of the sRNA population may enter the ILVs of the MVBs, possibly leading the subset onto the exosomal excretion pathway (Figure 2) [45], [46]. The ESCRT requirement for secretion of sRNA containing exosomes is uncertain since an alternative ceramide-dependent ILV formation mechanism, regulated by neutral sphingomyelinase2 activity, has been proposed [34], [47], [48]. It has been suggested that vesicle loading of sRNAs can depend on their binding to complementary mRNA, their sequence motifs, and their 3′ modifications. miRNAs in human primary T-lymphocyte–derived exosomes have been found to share four-base EXOmotifs, which bind the protein hnRNPA2B1 after its sumoylation. This ribonucleoprotein-complex is sorted into the MVB ILVs, subsequently secreted as exosomes [49]. sRNAs in exosomes are not only protected by a membrane. In the mammalian bloodstream, sRNAs in exosomes have been found to form a complex with Ago2 [33], as has been found for sRNA not enveloped by a membrane [37].

RNA signals in extracellular vesicles are envisaged to enter target cells in one of two ways. The intact vesicle can be endocytosed at the plasma membrane, after which the RNA will end up being behind two membranes in an endosome (Figure 2). RNA escape to the cytosol will require a fusion of the two membranes by an unknown mechanism. Alternatively, the extracellular vesicles can fuse directly with the plasma membrane and thereby release the RNA into the cytosol. This process is also poorly understood. Intracellularly, membrane fusion processes are mediated by SNARE proteins. However, fusion of extracellular vesicles to plasma membranes will require other fusogenic proteins. This process will be similar to membrane fusions occurring, for instance, during oocyte fertilization, infection by membrane enveloped viruses, and cell–cell fusion events. A number of extracellular fusogenic proteins, such as syncytin and AFF-1, have been implicated in these fusion processes [50], [51], and it will be interesting to learn about the role of such proteins in the fusion of RNA-carrying exosomes and shedding vesicles with target cells. Fusogenic proteins may also mediate fusion between the two membranes of the endosome resulting from endocytosis of a vesicle.

## Trans-kingdom RNA-Transfer

RNA secretion is believed to generate the extracellular RNA that is transported between hosts and parasites. For instance, *T. cruzi*–produced vesicles are loaded with an Argonaute protein and sRNAs, which potentially influence host gene expression [14]. Extracellular vesicular transport of sRNA is a candidate mechanism to facilitate trans-kingdom RNA transfer between other species as well. Plant leaves attacked by the powdery mildew fungus deliver both shedding vesicles and exosomes at the fungal attack site, and interference of the latter hampers plant defense [52]–[54]. This supports a possible role for vesicular transport of the RNA silencing signal and suggests a means of RNA delivery not only during HIGS but also for wild-type plants to transfer RNA to the fungus as a defense strategy [18], [54], [55].

Trans-kingdom RNAi could also depend on transfer of non-vesicular RNA. However, to our knowledge, functional homologs of SID-1 and SID-2, for instance, or an alternative direct RNA uptake system, have not been described in plants. Therefore, given the accumulation of vesicular material in the interphase between the plant and pathogen, we deem it likely that the dissemination of gene silencing RNA during HIGS in plants relies on vesicle-mediated transport, much like in mammalian circulation, where the spread of membrane-enveloped endogenous miRNA signals through the bloodstream requires the selective uptake of these signals by the recipient cells [18], [52] (Figure 2).

After entering the target cell, it is likely that sRNAs will make use of the RNAi-machinery of that cell. For instance, when the fungus *B. cinerea* exploits sRNAs to silence defense genes in Arabidopsis and tomato, this process is dependent on plant AGO1 (Figure 2) [23]. This protein controls the cytosolic RNAi pathways, suggesting target mRNA cleavage or translational inhibition. However, as mentioned before, it has been shown that sRNAs can reduce gene expression in species that lack the canonical RNAi-mechanisms. *P. falciparum* does not possess homologs of AGO and DCR proteins [31], but the translocated miRNAs from human cells were found to form chimeric dsRNAs with *P. falciparum* transcripts, inhibiting translation (Figure 2) [16]. In plants, sRNA signals that are mobile through the phloem can induce marked reductions in gene expression in remote target cells, even though their concentration is very low (down to 10 parts per million) [56]. This is most likely also the case in trans-kingdom transfer of sRNA, since large-scale transfer of sRNA seems not feasible. Studies of long-distance signaling in plants using grafting revealed a necessity for the RNA-dependent RNA-Polymerase (RdRP), RDR6, in sRNA recipient cells, which is thought to amplify the incoming silencing signal [57], [58]. It is plausible that in trans-kingdom transfer, sRNA signals will also be amplified in their target cells to be able to induce gene silencing. It has been suggested that organisms ingesting plant material that contains sRNAs amplify these signals in the cells lining the digestive gut [30]. However, this might not be achieved using RdRP in all species. For instance, insects do not possess this enzyme [59], but they can still be affected by HIGS [24], [30]. Therefore, they are likely to have a different system to amplify incoming RNA signals.

Direct evidence remains, showing that trans-kingdom RNAi also can feed into nuclear chromatin-based silencing pathways. Yet an enduring silencing effect has been recorded [26], which might suggest such chromatin-based mechanisms can be activated.

## sRNA Sequence-Complementarity Requirements

In order to have efficient gene silencing in the target organism, the delivered sRNA signals should meet the sequence-complementarity requirements specific to the receiving cell. These requirements vary between different kingdoms, for instance, being less stringent in animals than in plants, and the requirements also vary according to the silencing pathway [4], [60], [61]. This is essential when designing hairpin constructs to target a transcript in an interacting organism. Generally, these constructs are made with complete sequence identity, but the complementarity requirements are important for the prediction of off-target transcripts. Natural sRNAs able to target transcripts in a trans-kingdom manner, such as those identified by Weiberg et al. [23], obviously obey the stringency criteria of the target kingdom, which is, in this case, plants. Here, the *B. cinerea* fungus produces 73 sRNAs with potential targets in Arabidopsis and tomato. Of these, three 21-nt retrotransposon-derived siRNAs target four plant transcripts important for pathogen defense, despite three to five mismatches. This increases the chance of sRNAs to be functional in an interacting organism and leads to speculation on whether such mechanisms have arisen fortuitously. Since the presence of matching sRNAs can provide a clear selective advantage, it is likely not to be a random occurrence. Furthermore, the retrotransposon origin of these sRNAs could indicate that these elements contribute to relatively rapid evolution of the sequence of host-directed sRNA, which is an advantage in the host–pathogen arms race.

## Common Emerging Concepts

Trans-kingdom RNA signaling is now a documented phenomenon with intriguing implications for our understanding of biological interactions. Similar to ideas proposed by Sarkies and Miska on “social RNA” [40], the presented evidence for trans-kingdom RNAi suggests that genetic interaction between organisms at the RNA level is common. Organisms become genetically programmed according to endogenous and environmental input. Hitherto, we have known these to include physical and chemical stimuli from other organisms. However, now we see that genetic programming also is influenced by genetic stimuli in the form of environmental RNA. Obvious biological niches where such RNA communication could be prolific would be in the soil, where evidence for this already has been seen between plants and symbiotic mycorrhizal fungi [18], and on the skin and in the gut of animals.

Even though convincing, the data available for trans-kingdom RNAi are fragmented and mostly based on input sRNA sequences and phenotypic effects in the receiving organisms. No example provides information for the whole RNA signaling chain, which conceptually should involve sRNA production, secretion, uptake, perception, amplification and manifestation. For each of these steps, evidence for alternative mechanisms has been presented. However, between eukaryotic organisms with the canonical RNAi mechanisms intact, it appears from the evidence available that most trans-kingdom RNAi signaling follows the route: (1) sRNA production, (2) RNA secretion in MVB-based exosomes, (3) fusion of RNA containing exosomes to the plasma membrane, (4) RdRP-dependent amplification integrated with transcript cleavage and inhibition (Figure 2). We think of alternative mechanisms for each step as variations of these.

## Perspectives

It is now documented by many examples that eukaryotic organisms of different kingdoms exchange RNA sequences as signals affecting gene expression, and we may only have seen the tip of the iceberg of this phenomenon. Future studies that investigate the mechanisms of this trans-kingdom RNA transfer more systematically, will most likely identify “the usual suspects” of the canonical silencing machinery (e.g., Dicers and Argonautes) as being required for production of mobile RNA and the hijack of the target-cell RNA silencing machinery. The biggest revelations may come in the form of factors that are involved in RNA export from the producing cell, its physical extracellular transport and its import into target cells. These mechanisms are very enigmatic at this point, and we can only speculate by comparison to analogous phenomena within organisms. So far, HIGS has focused on the function of target genes, but we foresee that it could be used to dissect the process of trans-kingdom RNA-silencing transfer by setting up carefully designed screens. We think that HIGS systems, in which plant expression of hpRNA directed against genes in the pathogen, hold a big promise as a mechanism for pest control, since the system has been described to work effectively in an increasing number of species [15], [18], [21], [22], [25]. Targeting of essential invader genes would appear to be advantageous to current exploitation of endogenous defense mechanisms in that it should not influence other processes in the host, and that the invader may have larger difficulty in overcoming it.
